# Supplementary material for: Valence and spin states of iron are invisible in Earth’s lower mantle
Source: Nat Commun. 2018 Mar 29;9:1284. doi: 10.1038/s41467-018-03671-5 (PMC5876394; doi:10.1038/s41467-018-03671-5)
Supplement: Supplementary file 1 — Supplementary information(PDF 9514 kb) [file 41467_2018_3671_MOESM1_ESM.pdf]

Valence and spin states of iron are invisible in Earth's lower mantle

Liu et al.

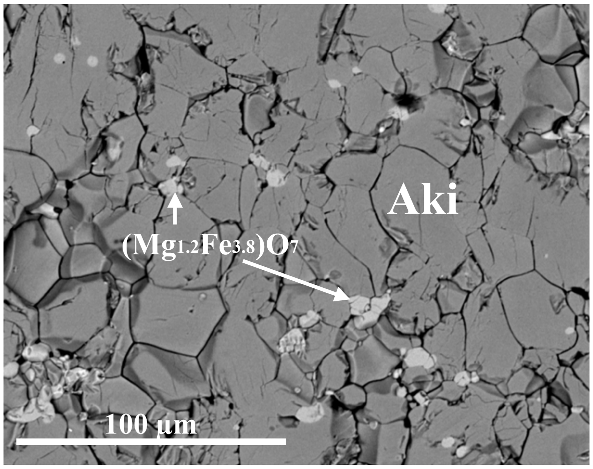

Supplementary Figure 1: Back-scattered-electron image of recovered run product from multi-anvil synthesis at 24 GPa and 1973 K. The major phase is $Mg_{0.46}Fe_{1.04}Si_{0.49}O_3$ akimotoite and the bright minor phase is $(Mg_{1.2}Fe_{3.8})O_7$.

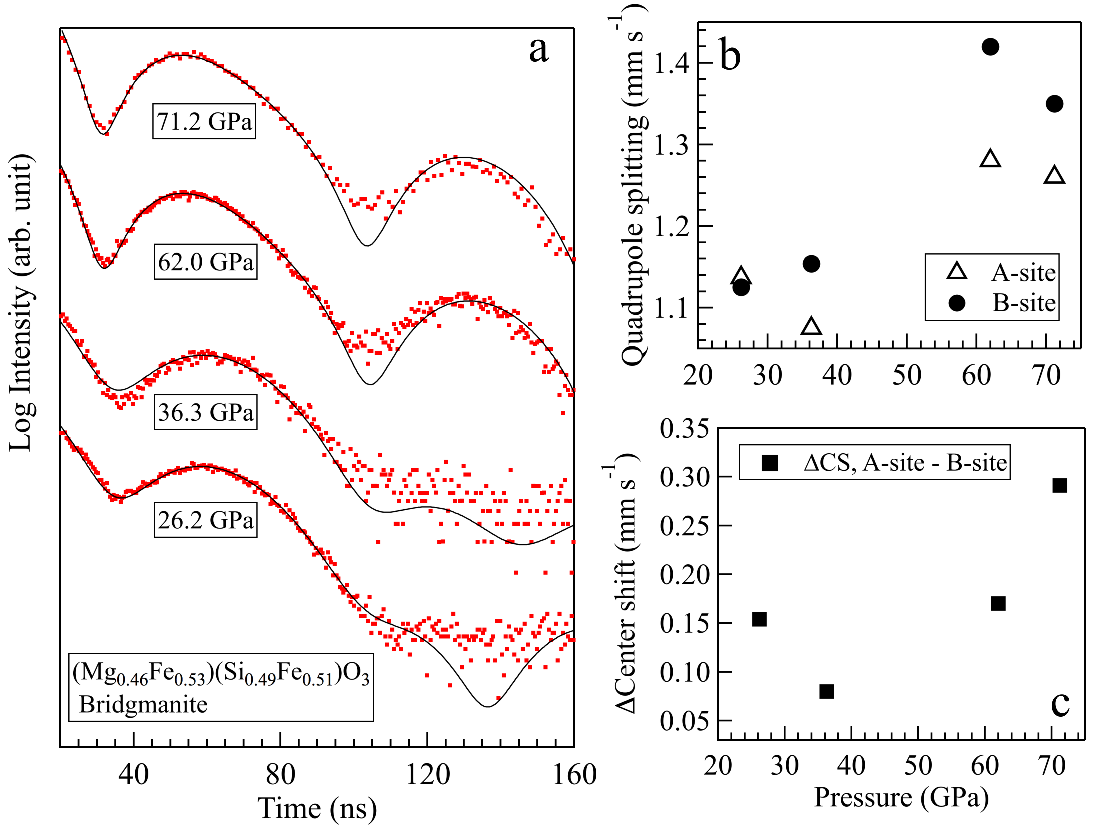

Supplementary Figure 2: Hybrid-mode synchrotron Mössbauer results for $(Mg_{0.46}Fe^{3+}_{0.53})(Si_{0.49}Fe^{3+}_{0.51})O_3$ bridgmanite. (a): The hybrid-mode synchrotron Mössbauer spectra (red dots) of $(Mg_{0.46}Fe^{3+}_{0.53})(Si_{0.49}Fe^{3+}_{0.51})O_3$ bridgmanite between 26.2 and 71.2 GPa at room temperature. The black curves are fits obtained using a two-site model with site weights fixed at 50:50. (b): Quadrupole splitting (QS) of the two sites as a function of pressure. Across the spin transition, QS increases by 0.1-0.2 mm/s in the A-site and 0.2-0.3 mm/s in the B-site. (c): Relative center shift (ΔCS=A-site – B-site) as a function of pressure at 300 K. Across the spin transition, ΔCS increases by 0.1-0.2 mm/s.

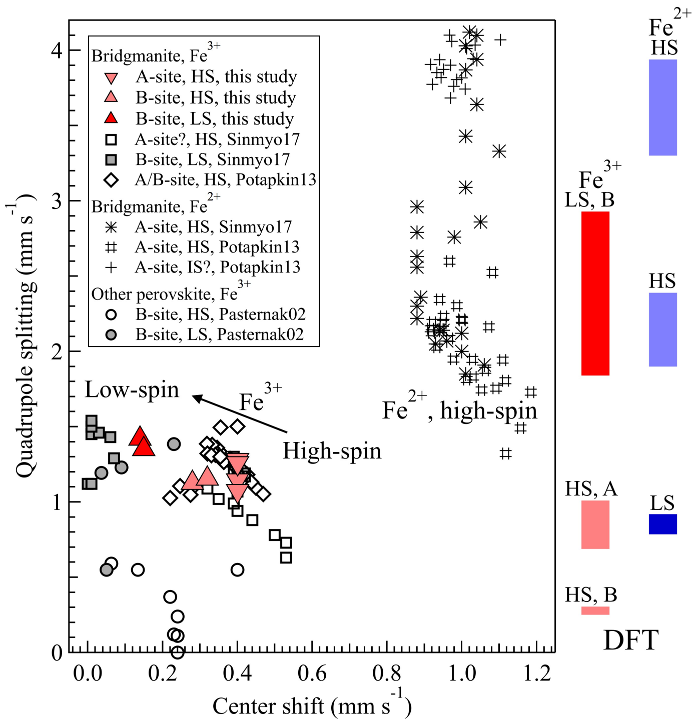

Supplementary Figure 3: A summary of quadrupole splitting and center shift of bridgmanite samples at high pressures. Except this study, all the literature data shown here were obtained by synchrotron-based energy-domain Mössbauer spectroscopy, which can allow more unambiguous fitting relative to nuclear forward scattering, particularly for CS, but sacrifices precision. The arrow indicates the trend of changes in QS and CS for spin transitions of B-site Fe^{3+} in bridgmanite. The density functional theory results for Fe species with different valence and spin state are listed on the right (ref. 1,2). Experimental data from $(Mg_{0.46}Fe^{3+}_{0.53})(Si_{0.49}Fe^{3+}_{0.51})O_3$ bridgmanite in this study are marked as triangles, where A-site HS Fe^{3+} (down-pointing) and B-site HS Fe^{3+} (up-pointing) are in light red, and B-site LS Fe^{3+} (up-pointing) is in red. CS of A-site HS Fe^{3+} is assumed to be 0.4 mm/s and CS of B-site Fe^{3+} is calculated based on ΔCS from Mössbauer results. Data from ref. 3: A-site HS Fe^{2+} (stars); B-site LS Fe^{3+} (gray squares). The open squares were described as A-site HS Fe^{3+} by ref. 3, but they are likely to be B-site HS Fe^{3+} as the lower-pressure counterpart of the B-site LS Fe^{3+}. Data from ref. 4: A-site HS Fe^{2+} (pounds), A/B-site HS Fe^{3+} (open diamonds). Ref. 4 attributes the extremely large QS component (crosses) to Fe^{2+} intermediate-spin state, but this site could also be ascribed to A-site HS Fe^{2+} with lattice distortion[1,2]. In comparison, the open and gray circles are for B-site HS and LS Fe^{3+} in other perovskites[5], respectively.

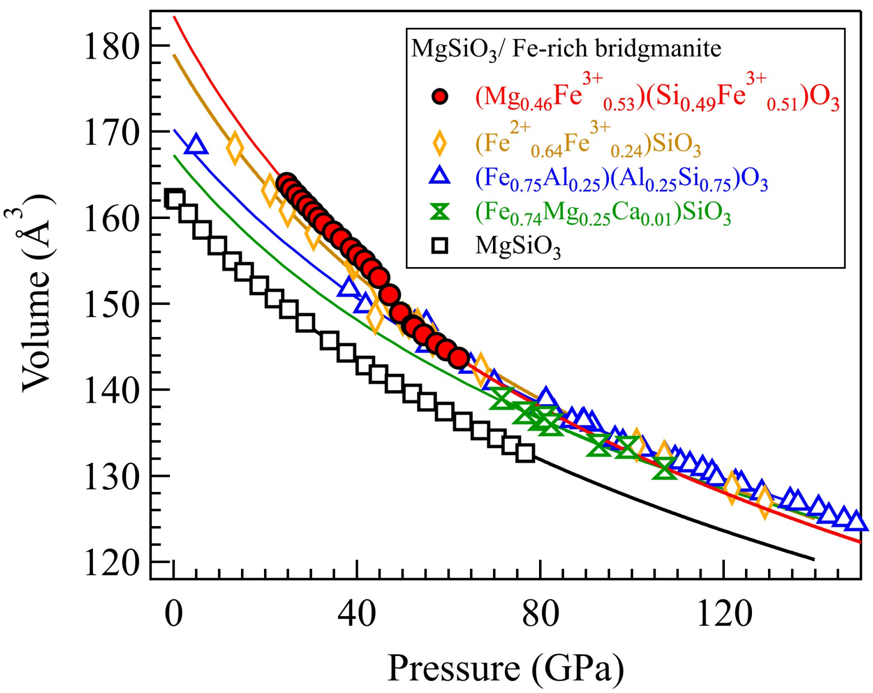

Supplementary Figure 4: Unit cell volumes on compression and decompression for bridgmanite with different incorporation of Fe. The symbols are observed data and the curves are the corresponding 2nd BM-EoS fits to the data. Data source: $(Mg_{0.46}Fe^{3+}_{0.53})(Si_{0.49}Fe^{3+}_{0.51})O_3$ (red circles with black rim): this study; $(Fe^{2+}_{0.64}Fe^{3+}_{0.24})SiO_3$ (yellow open diamonds): ref. 6; $(Fe_{0.75}Al_{0.25})(Al_{0.25}Si_{0.75})O_3$ (blue open triangles): ref. 7; $(Fe_{0.75}Mg_{0.25}Ca_{0.01})O_3$ (green open hourglasses): ref. 8; $MgSiO_3$ (black open squares): ref. 9.

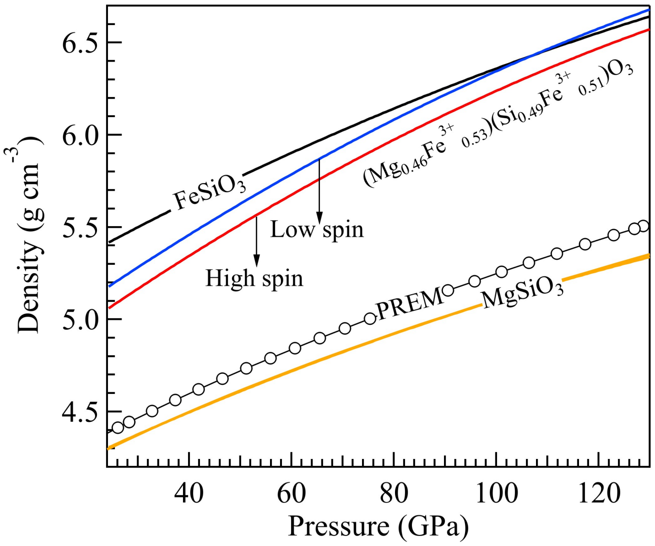

Supplementary Figure 5: Calculated density profiles of four bridgmanite compositions along mantle geotherm. The thermoelastic parameters for $MgSiO_3$ and $FeSiO_3$ end members are from ref. 10. For $(Mg_{0.46}Fe^{3+}_{0.53})(Si_{0.49}Fe^{3+}_{0.51})O_3$ high spin and B-site low spin bridgmanite samples, the 300-K elastic parameters are from this study and the parameters for calculating Mie-Grüneisen-Debye thermal pressure are adopted from $AlAlO_3$ bridgmanite reported in ref. 10. The geotherm is from ref. 11. The preliminary reference Earth model (PREM)[12] is plotted for comparison (black curve with open circles).

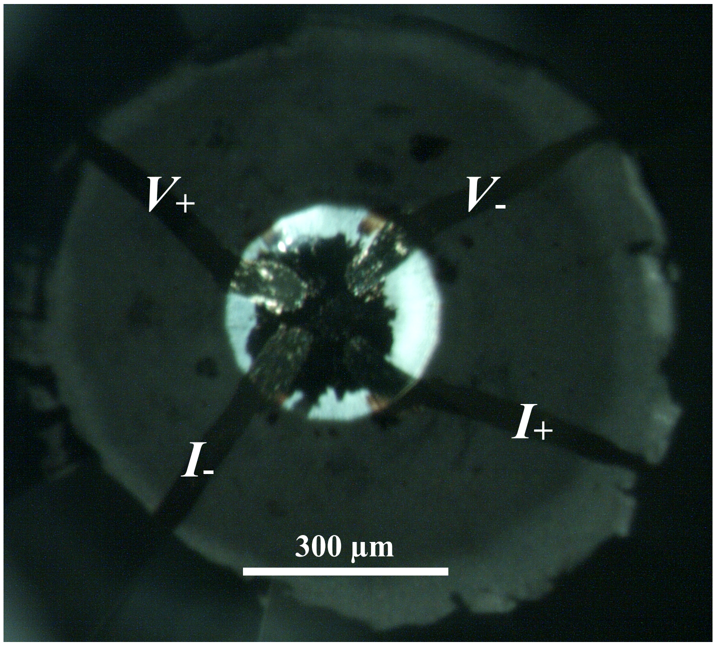

Supplementary Figure 6: Optical image of bridgmanite sample during electrical resistance measurements by four-point-probe method in a DAC at 64 GPa. Four Au leads were mounted to $(Mg_{0.46}Fe^{3+}_{0.53})(Si_{0.49}Fe^{3+}_{0.51})O_3$ Bdg sample. Current was supplied through two adjacent Au leads (marked "*I*") while the other two leads (marked "*V*") measured the corresponding voltage. Electrodes were insulated from stainless steel gasket by cBN powder.

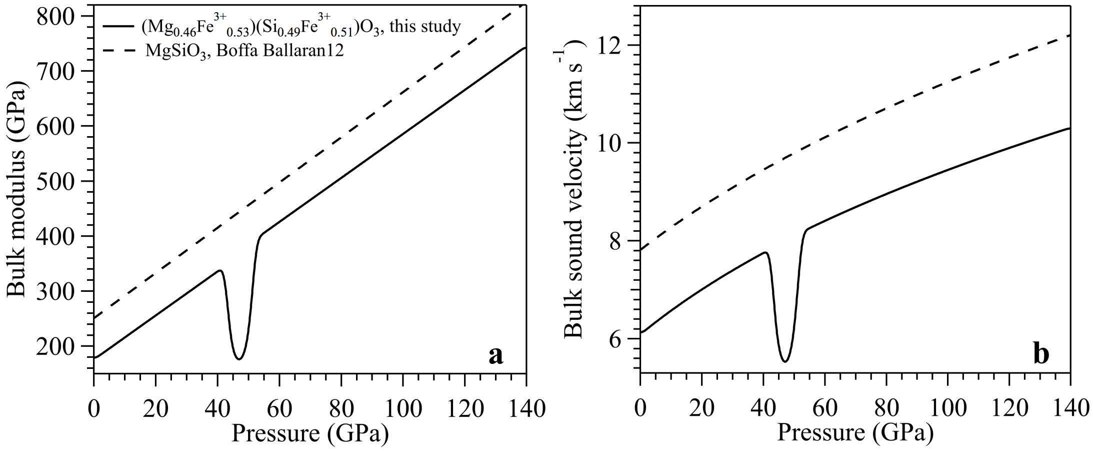

Supplementary Figure 7: Bulk modulus (a) and bulk sound velocity (b) profiles of two bridgmanite samples at 300 K. The solid curves are for $(Mg_{0.46}Fe^{3+}_{0.53})(Si_{0.49}Fe^{3+}_{0.51})O_3$ bridgmanite from this study and the dashed curves are for $MgSiO_3$ bridgmanite[9] at 300 K. The bulk modulus across the spin-transition pressures is modeled following ref. 13.

Supplementary Table 1. Mössbauer parameters obtained from fits to hybrid-mode synchrotron nuclear forward scattering data for $(Mg_{0.46}Fe^{3+}_{0.53})(Si_{0.49}Fe^{3+}_{0.51})O_3$ bridgmanite at 300 K. Fit parameters include quadrupole spitting (QS) for A-site and B-site Fe^{3+} and relative center shift difference between the two sites (ΔCS). Weights of the two sites are fixed at 50% each.

P (GPa)	QS (mm s^{-1}) A-site	QS (mm s^{-1}) B-site	ΔCS (mm s^{-1}) A-site - B-site
26.2(3)	1.14(1)	1.13(1)	0.15(1)
36.3(4)	1.07(2)	1.15(1)	0.08(1)
62.0(2)	1.28(2)	1.42(1)	0.17(1)
71.2(4)	1.26(1)	1.35(1)	0.29(1)

Supplementary Table 2. Observed volume and unit-cell parameters of $(Mg_{0.46}Fe^{3+}_{0.53})(Si_{0.49}Fe^{3+}_{0.51})O_3$ bridgmanite at 300 K. Au was used as the pressure standard (14). Numbers in parentheses are uncertainties on the last digits.

P (GPa)	*a* (Å)	*b* (Å)	*c* (Å)	V ($Å^3$)
24.7(1)	4.740(9)	4.974(9)	6.956(10)	164.0(5)
25.7(1)	4.729(9)	4.970(9)	6.950(10)	163.3(5)
26.9(1)	4.719(8)	4.967(9)	6.939(10)	162.7(5)
28.0(1)	4.710(8)	4.964(9)	6.929(10)	162.0(5)
29.2(1)	4.703(8)	4.958(9)	6.920(10)	161.4(5)
30.4(1)	4.694(8)	4.952(9)	6.911(10)	160.6(5)
31.6(1)	4.687(9)	4.946(9)	6.901(10)	160.0(5)
32.8(1)	4.679(9)	4.940(9)	6.892(10)	159.3(5)
34.8(1)	4.667(9)	4.934(9)	6.876(10)	158.3(5)
36.6(1)	4.657(9)	4.929(9)	6.862(10)	157.5(5)
38.7(2)	4.644(9)	4.921(10)	6.846(10)	156.5(5)
40.1(2)	4.635(9)	4.915(10)	6.834(10)	155.7(5)
41.6(2)	4.626(9)	4.912(10)	6.822(10)	155.0(5)
43.1(2)	4.617(9)	4.901(10)	6.808(10)	154.0(5)
44.8(2)	4.606(9)	4.894(10)	6.790(10)	153.0(5)
47.1(2)	4.590(9)	4.869(10)	6.758(10)	151.0(5)
49.5(2)	4.571(9)	4.851(10)	6.716(10)	148.9(5)
52.0(2)	4.556(10)	4.839(11)	6.690(11)	147.5(5)
52.5(2)	4.554(10)	4.837(11)	6.688(11)	147.3(5)
54.6(2)	4.544(10)	4.832(12)	6.670(11)	146.4(5)
57.4(2)	4.529(11)	4.827(13)	6.651(13)	145.4(6)
59.5(2)	4.521(12)	4.820(13)	6.637(13)	144.6(6)
62.2(2)	4.510(13)	4.817(14)	6.616(15)	143.7(7)

Supplementary Table 3. Birch-Murnaghan equation of state parameters obtained by fitting volume data for $(Mg_{0.46}Fe^{3+}_{0.53})(Si_{0.49}Fe^{3+}_{0.51})O_3$ bridgmanite at 300 K. *P* is pressure, V_0 is ambient-pressure volume, K_0 is 1-bar bulk modulus; K_0' is the pressure dependence of bulk modulus at 300 K.

$(Mg_{0.46}Fe^{3+}_{0.53})(Si_{0.49}Fe^{3+}_{0.51})O_3$	*P* (GPa)	V_0 (Å^3)	K_0 (GPa)	K_0'
High spin	24.7-43.1	183.5(3)	176(2)	4(fixed)
Low spin	52.5-61.4	178.5(7)	186(5)	4(fixed)

Supplementary Table 4. Electrical conductivity measured in ($Mg_{0.46}Fe^{3+}_{0.53}$)($Si_{0.49}Fe^{3+}_{0.51}$)O_3 bridgmanite at 300 K. *P* is pressure, σ is electrical conductivity. Run 2 is a successive compression experiment after the pressure is released after run 1.

run1		run2	
P (GPa)	Log[σ (S m^{-1})]	*P* (GPa)	Log[σ (S m^{-1})]
31.2(4)	-2.37	30.7(3)	-2.51
33.3(4)	-2.31	32.3(3)	-2.35
35.1(5)	-2.25	34.7(3)	-2.28
38.1(3)	-1.99	39.7(2)	-2.00
40.8(3)	-1.99	46.0(3)	-1.81
43.0(3)	-2.01	50.2(3)	-1.70
43.9(3)	-1.96	51.9(2)	-1.34
45.6(3)	-1.92	56.0(3)	-1.17
48.2(2)	-1.83	58.0(4)	-1.07
51.2(3)	-1.71	60.9(2)	-0.93
54.2(3)	-1.40	63.9(6)	-0.83
57.6(4)	-1.10	65.9(7)	-0.75
60.3(2)	-0.95		
64.8(3)	-0.81		

Supplementary References

1. Bengtson, A., Li, J. & Morgan, D. Mossbauer modeling to interpret the spin state of iron in (Mg,Fe)SiO_3 perovskite. *Geophys. Res. Lett.* **36,** (2009).
2. Hsu, H., Blaha, P., Cococcioni, M. & Wentzcovitch, R. M. Spin-State Crossover and Hyperfine Interactions of Ferric Iron in $MgSiO_3$ Perovskite. *Phys. Rev. Lett.* **106,** (2011).
3. Sinmyo, R., McCammon, C. & Dubrovinsky, L. The spin state of Fe^{3+} in lower mantle bridgmanite. *Am. Mineral.* **102,** 1263–1269 (2017).
4. Potapkin, V. *et al.* Effect of iron oxidation state on the electrical conductivity of the Earth's lower mantle. *Nat. Commun.* **4,** (2013).
5. Pasternak, M. P., Xu, W. M., Rozenberg, G. K. & Taylor, R. D. Electronic, magnetic and structural properties of the $RFeO_3$ antiferromagnetic-perovskites at very high pressures. *Perovskite Mater.* **718,** 15–24 (2002).
6. Ismailova, L. *et al.* Stability of Fe,Al-bearing bridgmanite in the lower mantle and synthesis of pure Fe-bridgmanite. *Sci. Adv.* **2,** e1600427 (2016).
7. Dorfman, S. M., Shieh, S. R., Meng, Y., Prakapenka, V. B. & Duffy, T. S. Synthesis and equation of state of perovskites in the (Mg, Fe)$_3Al_2Si_3O_{12}$ system to 177 GPa. *Earth Planet. Sci. Lett.* **357,** 194–202 (2012).
8. Dorfman, S. M., Meng, Y., Prakapenka, V. B. & Duffy, T. S. Effects of Fe-enrichment on the equation of state and stability of (Mg,Fe)SiO_3 perovskite. *Earth Planet. Sci. Lett.* **361,** 249–257 (2013).
9. Ballaran, T. B. *et al.* Effect of chemistry on the compressibility of silicate perovskite in the lower mantle. *Earth Planet. Sci. Lett.* **333,** 181–190 (2012).

10. Stixrude, L. & Lithgow-Bertelloni, C. Thermodynamics of mantle minerals - II. Phase equilibria. *Geophys. J. Int.* **184,** 1180–1213 (2011).

11. Brown, J. M. & Shankland, T. J. Thermodynamic parameters in the Earth as determined from seismic profiles. *Geophys. J. R. Astron. Soc.* **66,** 579–596 (1981).

12. Dziewonski, A. M. & Anderson, D. L. Preliminary Reference Earth Model. *Phys. Earth Planet. Inter.* **25,** 297–356 (1981).

13. Wentzcovitch, R. M. *et al.* Anomalous compressibility of ferropericlase throughout the iron spin cross-over. *Proc. Natl. Acad. Sci. U. S. A.* **106,** 8447–8452 (2009).

14. Fei, Y. W. *et al.* Toward an internally consistent pressure scale. *Proc. Natl. Acad. Sci. U. S. A.* **104,** 9182–9186 (2007).
